# Management of Refractory Menstrual Bleeding in an Adolescent with Glanzmann Thrombasthenia: A Case Report and Review

**DOI:** 10.1155/2020/8848763

**Published:** 2020-09-30

**Authors:** Luke J. King, Jessie Huff, Deidre Heber, Michal Ann Miller, Brytanie Marshall

**Affiliations:** ^1^Obstetrics & Gynecology, Geisinger Medical Center, USA; ^2^Pediatric Hematology/Oncology, Geisinger Medical Center, USA

## Abstract

Glanzmann Thrombasthenia is a rare bleeding disorder causing life-threatening bleeding at menarche in the adolescent female. Bleeding often necessitates admission and multiple blood transfusions. Due to the rarity of the disease, management of acute bleeding in new-onset menarche poses a particular challenge. A 12-year-old menarchial female had persistent vaginal bleeding despite multiple treatment modalities including aminocaproic acid, recombinant factor VIIa, intravenous estrogen, and gonadotropin receptor hormone agonists. Although the standard treatment of bleeding in patients with GT is primarily rFVIIa, new-onset menstrual bleeding in conjunction with an immature hypothalamic-pituitary-ovarian axis often requires expanding treatment to include multiple drug modalities. In our case, a two-step approach was necessary. The first is targeting the cessation of the first menses. The second is optimizing ongoing management of long-term control of heavy menstrual bleeding to achieve amenorrhea, prevent further hospital admissions, and avoid recurrent transfusions.

## 1. Introduction

Glanzmann Thrombasthenia (GT) is a rare inherited autosomal recessive bleeding disorder characterized by abnormal platelet aggregation. Platelet dysfunction is secondary to a defect in the platelet membrane receptor IIB/IIIa (Integrin *α*IIb*β*3), preventing platelet activation in response to agonists such as ADP, collagen, or thrombin [1–3]. The frequency of the disease is extremely rare with an incidence of 1 per million [3]. The prevalence of the disease, however, is higher in communities where consanguinity is common, as demonstrated in Jewish and Southern Indian populations [2].

Diagnosis of GT is often made in childhood due to recurring episodes of bleeding including: epistaxis, mucosal and dental bleeding, easy bruising, and gastrointestinal bleeding. More serious bleeding episodes—such as central nervous system bleeding, hemoperitoneum, hemopericardium, and bleeding from trauma—are less common but carry a higher rate of mortality [2, 4]. Platelet transfusion remains the standard treatment for nonsurgical bleeding in patients with GT [4]. The use of recombinant factor VIIa (rFVIIa) has been proven to be effective in managing nonsurgical bleeding that is refractory to platelet transfusions [4, 5].

Adolescent females with GT often suffer from heavy menstrual bleeding (HMB), clinically defined as blood loss of more than 80 mL per cycle. The prevalence of HMB in females with GT exceeds 9 per 10 cases and can severely affect their quality of life leading to multiple hospital admissions and blood transfusions [6]. The treatment options of GT-related HMB are largely based on case studies and expert opinion to date [7]. In adolescents, HMB is especially challenging to treat secondary to the immaturity of the hypothalamic-pituitary-ovarian (HPO) axis, as demonstrated in this case study. Currently, there is no standard treatment in this adolescent population. Thus, we provide a novel approach to the management of HMB in female adolescents with GT.

## 2. Case Presentation

A 12-year-old female with a known history of GT presented to the Emergency Department (ED) with a chief complaint of HMB. She was diagnosed with GT in infancy in setting of extensive bruising, recurrent mucosal bleeding, and persistent gastrointestinal bleeding. Prior to menarche, she had recurrent episodes of epistaxis and an episode of deep tissue hematoma that was managed outpatient with aminocaproic acid. She had two hospital admissions secondary to gingival and dental bleeding requiring treatment with intravenous recombinant rFVIIa and a tooth extraction. She had no need for major surgical procedures to date. She was followed by a Pediatric Hematologist for her condition but had not yet been evaluated by a Pediatric Gynecologist prior to menarche due to limited access.

Prior to presentation to the ED, she had experienced menarche followed by 24 days of HMB refractory to high-dose oral contraceptive taper (norgestimate-ethinyl estradiol 0.25 mg-25 mcg three times daily for three days, twice daily for two days, and daily thereafter). As bleeding continued, she was brought to the ED for further evaluation and admitted for management of acute blood loss anemia with a team of Pediatric Hematologists and Gynecologists.

Evaluation included a transabdominal pelvic ultrasound that revealed a thin endometrial stripe of 5 millimeters. She was started on intravenous estrogen therapy (intravenous conjugated estrogen 25 mg every four hours) and transitioned to oral estrogen (conjugated estrogen 2.5 mg every 4 hours) for a total of 48 hours. During this initial phase, the patient required 5 units of packed red blood cells due to acute blood loss anemia. Upon improvement of her vaginal bleeding similar to a light menses, she was transitioned to daily oral progesterone (norethindrone acetate 10 mg twice daily). Unfortunately, her HMB soon returned. Due to persistent bleeding refractory to the aforementioned hormonal therapy, she received leuprolide acetate (intramuscular 11.25 mg once) to suppress the HPO axis and induce a hypoestrogenic state. At this time, she was continued on oral progesterone (norethindrone acetate 10 mg twice daily) and aminocaproic acid to quell the ongoing bleeding and allow for leuprolide acetate to take therapeutic effect. Five days after administration of leuprolide acetate, the patient achieved amenorrhea and was discharged.

The patient had several weeks without vaginal bleeding and a norethindrone taper was attempted (5 mg daily for a week, 2.5 mg for a week, and then 2.5 mg every other day for a week). However, due to interval return of vaginal bleeding, a transabdominal pelvic ultrasound was repeated and revealed an 11 mm endometrial stripe. The norethindrone and aminocaproic acid was resumed. She continued treatment with intramuscular leuprolide acetate and norethindrone “add-back” therapy for a total of six months with successful amenorrhea. She was then transitioned to continuous combined oral contraceptive (norethindrone-ethinyl estradiol 1.5 mg-30 mcg once daily) as the leuprolide acetate treatments were concluded. Multiple attempts were made to taper the norethindrone “add-back” but bleeding returned. A final transabdominal pelvic ultrasound revealed a 5 millimeter endometrial stripe, and she has been maintained with continuous combined oral contraceptive and additional norethindrone as needed without recurrence of HMB. Patient consent as well as parental consent was obtained to publish the aforementioned case presentation.

## 3. Summary and Conclusion

Treatment of acute blood loss anemia in GT patients has been well studied in the literature. Platelet transfusion remains the standard therapy for nonsurgical episodes of bleeding (epistaxis, gastrointestinal bleeding, etc.) in GT patients. Transfusion of platelets, especially multiple transfusions, is not without risk.

Platelet immunization or refractoriness is a potential risk and is the most concerning life-long complication for GT patients receiving platelet transfusions. Approximately 17% of GT patients receiving leukoreduced platelet transfusions will develop antibodies against HLAs and have been associated with developing alloimmunization to future platelet transfusions. This poses a dilemma as many patients will require multiple transfusions in their lifetime, propagating further refractoriness to increasing number of transfusions [4]. Up to 98.2% of GT patients will report clinical symptoms of HMB according to Poon et al., and thus, we cannot rely solely on multiple blood product transfusions for bleeding control [4, 8]. Due to the rarity of the disease, there is no standardized approach to managing this high prevalence of HMB among adolescents with GT. Thus, we rely on case reports to help guide management.

In our patient's case, she did not receive a premenarchial Pediatric Gynecology consultation which may have proved useful in preparation for menarche and potentially reduced the morbidity associated with her first menses. She had failed routine hormonal therapy and required a novel approach of leuprolide acetate-induced suppression of the immature HPO axis with concomitant hormonal therapy to regulate her HMB and achieve amenorrhea. The use of leuprolide acetate is not currently FDA approved for the treatment of HMB in adolescents with bleeding disorders, though its benefits of producing a hypoestrogenic state proved beneficial to our patient's HMB refractory to hormonal therapy. The mechanism of action of gonadotropin-releasing hormone agonist (GNRH-a) involves an initial increase in endogenous estrogen that is then followed by a hypoestrogenic state. In our patient, the temporary increase in estrogen was the presumed cause of the thickened endometrial stripe found on ultrasound. Norethindrone was utilized to mitigate any additional bleeding caused by this effect. In the long term use of GNRH-a, caution should be exercised to avoid the additional risk of bone mineral density (BMD) loss with greater than 6 months of treatment. The use of norethindrone additionally also provides “add-back” therapy, to prevent the deleterious effect of GNRH-a on BMD [9]. As in our case, leuprolide acetate was limited to less than 6 months to prevent potential BMD.

Other methods have been described in the literature to achieve initial cessation of HMB in this patient population ranging from uterotonics such as methylergonovine maleate to uterine packing with tampons [10]. Lu and Yang found success with the levonorgestrel-releasing intrauterine system (LNG-IUS) in two 14-year-old patients with a history of HMB since menarche [6]. Due to higher rate of expulsion in women with bleeding disorders, Lee et al. recommend acute management of HMB prior to placement of the LNG-IUS [10–13]. Both cases required that the patient achieve lighter periods with hormonal therapy prior to insertion of the LNG-IUS. As was the case with this patient, she was initially a poor candidate for an intrauterine system as she had significant heavy bleeding at the time. After in-depth counseling with the patient and parents, they did not desire to pursue an intrauterine system. In the future, as her bleeding continues to be controlled, the use of the LNG-IUS should be revisited as a reasonable long-term option in the prevention of her HMB.

In the reproductive age woman, treatment of HMB with rFVIIa has been shown to be successful in several case studies. However, in the adolescent patient with HMB, rFVIIa is not sufficient in treating the underlying mechanism of anovulatory bleeding in the setting of an immature HPO axis [5]. To date, the only curative treatment is allogenic hematopoietic stem cell transplant. However, only limited cases have been reported in the literature with varying degrees of success [14]. Due to the complexity of this disease in a high-risk population, we provide a unique case of successful use of GNRH-a with transition to oral hormonal therapy to treat and prevent acute blood loss anemia in an adolescent with GT-related HMB.

## Abbreviations

ED: Emergency Department
FDA: Food and Drug Administration
GT: Glanzmann Thrombasthenia
GNRH-a: Gonadotropin-releasing hormone agonist
HPO: Hypothalamic-pituitary-ovarian
HMB: Heavy menstrual bleeding
LNG-IUS: Levonorgestrel-releasing intrauterine system
rFVIIa: Recombinant factor VIIa.
